# Journal Club is a way forward to adopt Evidence Based Practice among dental House Officers

**DOI:** 10.12669/pjms.38.1.4562

**Published:** 2022

**Authors:** Kiran Fatima Mehboob Ali Bana

**Affiliations:** Dr. Kiran Fatima Mehboob Ali Bana, BDS, MCPS-HCSM, MHPE Assistant Professor, Department of Medical Education (Dental College), Bahria Dental College Karachi, Bahria University of Health Sciences (BUHS), Karachi, Pakistan

**Keywords:** Critical appraisal, Evidence based practice, Healthcare research, Journal Club, Self-directed learning

## Abstract

**Objectives:**

To identify the improvement in knowledge, presentation skills, critical skills and self-directed learning process during Journal Club platform amidst dental house officers of 2018, 2019 and 2020 after completing one year house job training at Bahria dental College Karachi.

**Methods:**

This cross-sectional study was conducted from Dec- 2018 till Dec-2020. The six items were asked about perception of change for evidence-based process of (knowledge, cognitive, affective and participant domains) after completion of house job training by each cohort. The responses were noted on three point likert scale as agree, neutral and disagree. Total 150 questionnaires were distributed in three cohorts. The SPSS version 23 was used. P-value < 0.05 was considered as statistically significant.

**Results:**

Total n=145 house officers had completed the proforma with response rate of 96.65%. The mean age was 24.45 ± SD 0.63 among three groups. There were n=20(14%) males and n=125(86%) females. There was improvement found for knowledge acquisition about relevant literature search among all three groups. Regarding knowledge acquisition of bio-statistics; majority of subjects n=26 (52%) in 2020 group had reported no change and in 2019 cohort n=23(48%) were agreed. Majority n=21(44%) of house officers had reported no change when asked as JC helped in critical thinking in year 2019.

**Conclusion:**

Knowledge acquisition about relevant literature search, presentation and confidence skills were improved but no significant changes were found in knowledge of biostatistics and critical thinking skills. JC is a convincing platform to learn evidence-based process amid dental house officers.

## INTRODUCTION

Evidence-based practice has become the gold standard for optimal patient outcome and quality of care.^1^ To practice evidence- based protocols; healthcare personnel should be competent to practice evidence-based decision making. As reported by Melnyk et al; 2018^2^; an estimated 2344 nurses in USA rated themselves as less competent to practice evidence-based activities. Because they felt that they need training in basic elements of evidence-based process such as formulate a research question, methodology, searching for the current relevant literature; appraise critically the evidence, integrate evidence in clinical setting, compare and evaluate the outcomes. Evidence- based practice is incorporated in curriculum of medical, dental and nursing education to address current best practices in healthcare worldwide.^1^ Literature proposed that clinical competency is linearly associated with the evidence-based knowledge of research during graduate and post graduate training.^3^ Moreover; Journal Club is an effective tool to change learners approach towards critical appraisal skills and research process among Ph-D scholars in medical education.^4^ Discrepancies in research content and evidence based practice may contribute to insufficient knowledge, attitude and skills among nursing graduates.^2^ To overcome this; the implementation of journal club is a way forward to develop evidence-based competency during undergraduate level.^1^,^2^

Journal clubs have been used among professionals and students in both academic and clinical settings for more than 200 years from Osler to twitter.^5^ The Journal Club (JC) is organized as regular group meetings conducted inside or outside academic walls. JC enabled students and clinicians to apply evidence-based practices in their relevant field by critically analyzing the published literature. It encourages students to search evidence-based literature by searching relevant research, gathers data of the best practices, actively participating in discussion with peers and seniors to critically appraising the published literature and integrating evidence into practice.^6^ To bridge this gap of research evidence and clinical practice; the Journal club is a recognized platform according to Rosen & Ryan in 2019.^7^ In addition; presentation skills, confidence to appraise evidence, team building skills are enhanced among regular participants of JC by creating large community of evidence- based practitioners.^8^

For final year dental and medical students; it is vital to understand the importance of evidence-based practice and critically appraised the best evidences and innovations in clinical settings, when they are a step away from entering in their independent practice.^9^ It is reported that 70% of occupational therapy undergraduates received research training from graduate schools.^5^ On the other hand; 76% of undergraduates’ proposed that research component should be the incorporated in first two years of medical curriculum according to Ahmed. F et al.^10^ Evidence revealed that students are interested in research conduction but the main challenge is proper guidance and lack of facilities.^11^ In addition; it transforms students into self-directed learners.^12^,^13^

Journal Club is implemented among faculties of Bahria University Medical and Dental College (BUMDC) since its inception.^14^ JC is structured & planned educational activity. Its schedule is disseminated to all dental house officers at the beginning of their house job training. The ideology is to change of research culture of publish not perish among dental house officers. Hence; the rational of this study was to recognize the activity of journal club as a way forward to adopt current best practices among dental house officers. It was hypothesized that knowledge, critical appraisal skills and self-directed learning were enhanced by regularly attending the JC meetings. Thus; this study was aimed to identify the improvement in knowledge, presentation skills, critical skills and self-directed learning process during Journal Club platform amidst dental house officers of 2018, 2019 and 2020 after completing one year house job training at Bahria dental College Karachi.

## METHODS

The questionnaire study was conducted amid dental house officers of 2018, 2019 & 2020 at Bahria Dental College, Karachi from Dec- 2018 till Dec-2020. This project was implemented after obtaining ethical approval from ERC Ref-44/2018. Data collection was carried out at three point of time (for three years) after one group of house officer is about to complete their house job training. In this study three batches (2018-2020) of house officers were approached via non probability convenience sampling technique used in this study.

**Fig.1:**
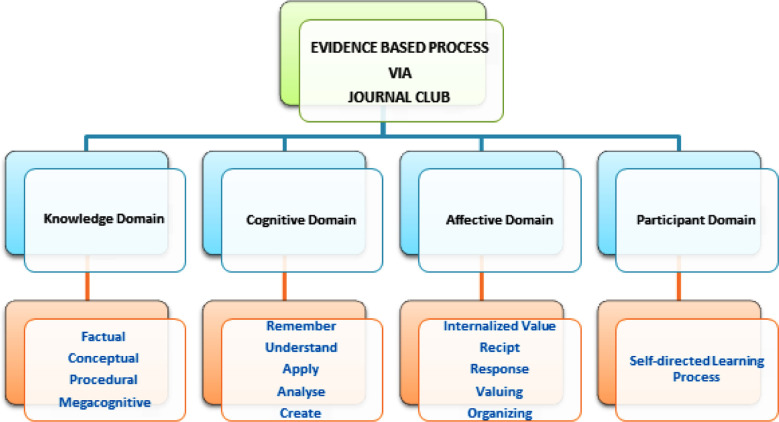
Evidence Based Practice.

Those house officers who had 90% attendance in JC meeting for one academic year were included after informed consent. The annual retake, absentees and irregular participants were excluded. The sample size was calculated by keeping the anticipated population as 150 for all three batches (50 participants per group) of dental interns on sample size formula N = Z2*P (I-P)/d2. The calculated sample size was 109. In year 2018 and 2019; the JC was conducted in walls of academia but for year 2020; JC was conducted online for six months due to lockdown period of COVID-19 as entire teaching was transformed into virtual mode to optimize time away from institute. The questionnaire was formulated with the help of literature search 14 and has two sections. Section one was about the demographic data such as age, gender and year of house job training. Section two has total eleven items to understand the research process such as JC helped in recall and recognition of literature, understanding of discussed article, encouraged in literature search , understanding of biostatistics, acquired knowledge for recent research , consult journal of repute , research motivation, enhanced presentation skills, confidence skills, critical thinking skills and self-directed learning. The responses were noted on three-point Likert Scale of 1=agree, 2=neutral and 3=disagree. The content validity of all eleven items was ascertained as all items were reflecting the content for improvement in evidence-based practice through journal club. The content validity was checked by five senior faculty members of community health sciences, having additional qualification in medical education; the items were rated as essential, useful and non-essential. More than 50% agreement for essential category ascertained to keep that item deemed important to understand research process at undergraduate level in final tool and remaining below agreement were discarded according to Content Validity Ratio (CVR) by; Lawshe’s Method 1975.^15^ Reliability was checked by conducting the pilot study among 10 house officers and this data was not added in final results. The items of recall and recognition of literature, understanding of discussed article, encouraged in literature search , acquired knowledge for recent research , consult journal of repute , research motivation were discarded from final questionnaire. The final questionnaire had demographic variables such as age, gender and year of house job training and section two had five items such as; two items for knowledge acquisition (to know relevant literature search and to understand biostatistics), third item on improvement in critical thinking and fourth item for enhancement in presentation skills and confidence. Last fifth item was improvement in self-directed learning-Fig-1. Total 150 questionnaires were distributed at the end of the dental house job training. The completed forms were entered in SPSS version 23. Normality of data was checked by sign-test. Hypothesis testing was performed by comparing median and means of all items. Kruskal wallis and one way ANOVA test was calculated to compare the median, Q1 and Q3 and for intergroup difference for all three years. P-value < 0.05 was considered as statistically significant.

## RESULTS

**Graph-1:**
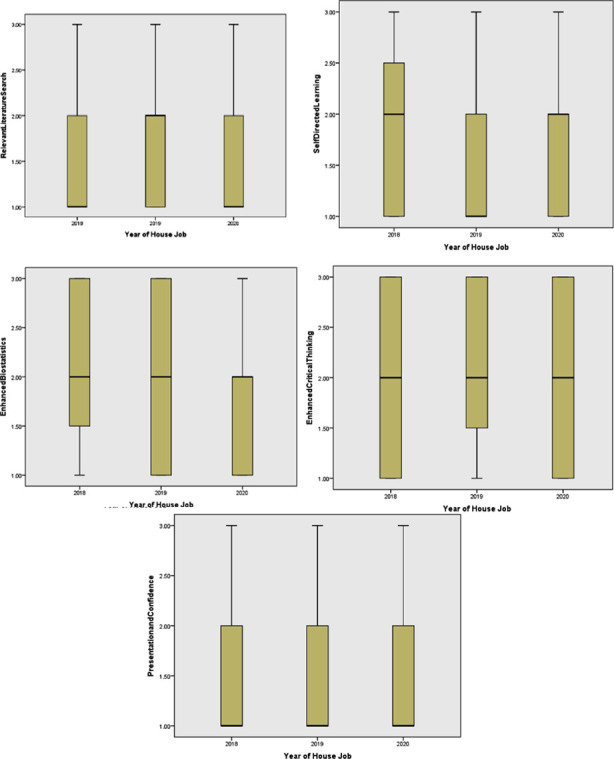
Comparing the responses among three groups of dental house-Officers.

The mean age of participants was 24.45 ± SD 0.63. There were 47 interns in year 2018, 48 in year 2019 and 50 in year 2020 from total 50 house officers per year; hence the response rate was 94% in 2018, 96% in year 2019 and 100% in year 2020. There was total n=20(14%) males and n=125(86%) females house officers altogether. Comparing the responses of JC; majority of house officers were agreed that knowledge was improved after JC activity and it helped in searching for relevant literature. Knowledge of bio-statistics was enhanced as majority of subjects n=26 (52%) in 2020 group had no change and in 2019 cohort around n=23(48%) was agreed that JC helped in understanding of Biostatistics. Majority n=21(44%) of dental interns responded neutrally when asked for improvement in critical thinking skills in year 2019. The presentation skills and confidence building were progressively increased as agreed by majority of subjects in 2018, 2019 & 2020 as n=34, n=32 & n=33 respectively. In year 2019; majority of participants n=27(56%) was agreed that self-directed learning was augmented -Graph-1; on the other hand, 2018 & 2020 groups had reported no change when interrogated about improvement in self-directed learning and significant difference was found while intergroup comparison of median at p-value of 0.036, Table-I. Therefore; the result of study was accepting the research hypothesis that knowledge domain of evidence-based practice was improved nonetheless no improvement was observed for critical appraisal skills and self-directed learning process by regularly attending the JC meeting.

**Table I:** Comparing Responses of Journal Club among three Cohorts of House Officers n=145.

Evidence based process items	2018 (N=47)			2019(N=48)			2020 (N=50)			P-Value

	Median	Q1	Q3	Median	Q1	Q3	Median	Q1	Q3	
1.Relevant Literature Search	1.0	1.0	2.0	2.0	1.0	2.0	1.0	1.0	2.0	0.373
2. Self-directed Learning	2.0	1.0	3.0	1.0	1.0	3.0	2.0	1.0	2.0	0.036

	Mean*		SD*	Mean*		SD*	Mean*		SD*	P-Value

3.Enhanced biostatistics knowledge	2.1		0.78	1.81		0.86	1.9		0.68	0.163
4. Enhanced critical thinking	2.04		0.8	2.06		0.75	1.9		0.82	0.866
5.Enhanced presentation Skills & confidence	1.3		0.67	1.4		0.68	1.48		0.73	0.792

Q_1:_ first quartile, Q_3_ third quartile: Kruskal Wallis test,

* Mean, SD: One way ANOVA.

Stem & leaf graph: 1=Agree, 2=Neutral, 3=Disagree

## DISCUSSION

Basic knowledge and principles of scientific inquiry, research design/process and practice in clinical settings are the evidence-based protocols for core research competency; and is mandated by worldwide accreditation Councils at exit level examination for dentists.^16^ Thereby; JC is a significant podium to achieve this outcome. Robust undergraduate knowledge, skills and attitudes of evidence-based practice has an impact over clinical practices.

In JC meeting; presenter has to present his/her evidence-based article in presentation format for 15 minutes. The presentation slide includes search strategy, introduction of topic and purpose of study; summarize research findings, methodology, applied statistical tests, critical appraisal, strengths and limitations and importance of topic in clinical setting. This presentation is followed by open ended discussion session.

The result of this study revealed that evidence-based process is well comprehended through regularly attending JC meeting. It was depicted that knowledge about search for relevant literature and biostatistics were enhanced by regularly attending JC meetings as agreed by 12(25%) and 23(48%) of house officers in batch 2018 & 2019 respectively but in 2020 group; n=26(52%) was responded neutrally that JC enhanced the knowledge of biostatistics. Albeit this, result might be due to the general perception of doctors as they like biology more than mathematics. These findings were comparable with the study of Szucs et al.^6^ They stated that participation in JC meetings positively enhanced competence in reading literature among students of various fields including occupational therapy and understanding of biostatistics was the biggest barrier identified by 62.1% of occupational therapy undergraduate to read literature.^6^

The presentation skills and confidence building were also improved as agreed by majority of subjects. These findings were complementary with the study of Szucs et al^6^; which proposed that students’ confidence of analyzing the evidence-based practice were augmented by regularly participating in JC meeting. Friesth et al.2020; suggested that presentation and writing skills were improved via reading quality literature and then present it confidently on JC forum.^9^

There was a progress found in critical thinking ability but no significant changes found in intergroup comparison. Critically appraising skills can be developed by making habit of reading updated evidence of best practices and then relate them in professional decision making in clinical setting.^17^ These results were contradictory with other studies which anticipated that discussion part and presentation format of JC are compelling to teach critical appraisal thinking among students.^18^ It is reported by MeLeods RS et al. in 2010 that teaching critical thinking skills among surgery residents; the face to face JC is more effective than virtual JC due to low attendance.^18^,^19^

This study acknowledged that there was improvement found in self-directed learning process among house officers but significant difference was found while intergroup comparison at p-value of 0.036. The participant domain of JC is to orient and encourage students to practice and become self-directed learners as presented in Fig.1. It is evident by Van Diggele et al.; that JC provide an innovative method of self-directed learning process especially in online environment via active participation.^12^ In year 2018 and 2019; the JC was conducted in walls of academia but for year 2020; JC was conducted virtually as emergency remote teaching for six months due to pandemic of COVID-19;^20^ the changing mode of JC in 2020 may empowered learners to take ownership of their learning process. By following “instructor scaffolded approach”^9^; the learner is able to understand the article in ‘chunks” which empower them to comprehend entire literature (from reading, search relevant literature, comprehend methodology, summarize the article in presentation format and respond during question answer session). This entire activity significantly indicated self-directed learning process and enhanced critical thinking process.^21^

JC activity is assuredly important during undergraduate level and the results would provide the way forward to make this podium more structured and fruitful to understand research process for evidence-based practice. This estimated the revision of JC activity which was indeed the strength of study.

### Limitations of the study:

Response bias is one of the strong limitations of this study. The cognitive and affective domains can be observed for research engagement of dental house officers in any research project by long term followup during undergraduate training period which is not observed in this study. JC activity in our context should be more interactive by leading discussion session on generalizability of evidence-based practice and by incorporating pre-posttest design^22^ and research and biostatistics workshops^23^. JC educational activity can provide quality teaching by attending it regularly, consistent advertisement of the event, structured and interactive speaker according to Khan et al. in 2017.^22^ This action may initiate thinking process among regular attendees and thus leads to read quality papers and think critically.

## CONCLUSION

Knowledge acquisition about relevant literature search was improved but no significant changes were found in knowledge of biostatistics and critical thinking skills. JC is a convincing platform to learn evidence-based process amid dental house officers.
